# Antimicrobial Resistance in New Zealand—A One Health Perspective

**DOI:** 10.3390/antibiotics11060778

**Published:** 2022-06-07

**Authors:** Isabelle Pattis, Louise Weaver, Sara Burgess, James E. Ussher, Kristin Dyet

**Affiliations:** 1 Institute of Environmental Science and Research Ltd., Christchurch 8041, New Zealand; louise.weaver@esr.cri.nz; 2 School of Veterinary Science, Massey University, Palmerston North 4442, New Zealand; s.burgess1@massey.ac.nz; 3 Department of Microbiology and Immunology, University of Otago, Dunedin 9054, New Zealand; james.ussher@otago.ac.nz; 4 Institute of Environmental Science and Research Ltd., Porirua 5022, New Zealand; kristin.dyet@esr.cri.nz

**Keywords:** AMR, resistance, antimicrobial, One Health, impacts, environmental AMR, New Zealand

## Abstract

Antimicrobial resistance (AMR) is an increasing global threat that affects human, animal and, often less acknowledged, environmental health. This complex issue requires a multisectoral One Health approach to address the interconnectedness of humans, animals and the natural environment. The prevalence of AMR in these reservoirs varies widely among countries and thus often requires a country-specific approach. In New Zealand (NZ), AMR and antimicrobial usage in humans are relatively well-monitored and -understood, with high human use of antimicrobials and the frequency of resistant pathogens increasing in hospitals and the community. In contrast, on average, NZ is a low user of antimicrobials in animal husbandry systems with low rates of AMR in food-producing animals. AMR in New Zealand’s environment is little understood, and the role of the natural environment in AMR transmission is unclear. Here, we aimed to provide a summary of the current knowledge on AMR in NZ, addressing all three components of the One Health triad with a particular focus on environmental AMR. We aimed to identify knowledge gaps to help develop research strategies, especially towards mitigating AMR in the environment, the often-neglected part of the One Health triad.

## 1. Introduction

Antimicrobial resistance (AMR) is a growing, serious global threat to human, animal and environmental health associated with antimicrobial use in humans and animals and waste discharges, including pharmaceutical wastewater [1,2,3]. AMR describes the adaptation of microorganisms, including bacteria, viruses, fungi and parasites, to grow in the presence of concentrations of antimicrobial agents that previously prevented growth or were lethal [4,5]. Development of AMR due to excessive or inappropriate antimicrobial consumption, poor choice of empiric antimicrobial therapy, combined with the spread of resistant bacteria and resistance-encoding mobile genetic elements (e.g., by water, food, poor sanitation, poor infection controls) accelerated by increased trade, travel and migration have made AMR a global health crisis [6,7].

There is international recognition that to comprehensively address the risk from AMR, a “One Health” approach across human health, animal health and the natural environment is necessary [8,9]. In 2015, a global action plan on AMR was endorsed by the World Health Assembly [8]. Consequently, countries worldwide developed frameworks outlining their individual responses to the global crisis. These frameworks naturally differ widely: in low- and middle-income countries, the focus is on challenges, such as weak regulation of production and sale of antimicrobials, whereas in high-income jurisdictions and countries such as the European Union (EU) and New Zealand (NZ), frameworks and action plans [10,11] include goals such as strengthening knowledge about AMR through research and surveillance, improving public awareness of AMR through communication and education, and aiming to improve infection prevention and control, mostly working towards “best practice”. To the best of our knowledge, the only action plan specifically addressing the environmental aspect in sufficient detail is the “European One Health Action Plan Against Antimicrobial Resistance” [11], which acknowledges the natural environment as a contributor to the development and spread of AMR, especially in high-risk areas. These high-risk areas include, but are not limited to, human, animal, and antimicrobials manufacturing waste. In NZ, compared to other countries, the burden of AMR is still relatively low, but resistance in a range of pathogens is increasing [12,13]. The “New Zealand National AMR Action Plan” includes five objectives: to improve awareness and understanding of AMR; to strengthen the knowledge and evidence base; to improve infection prevention and control measures; to optimise antimicrobial use in humans, animals and plants (antimicrobial stewardship); and to establish clear governance and collaboration to sustainably minimise AMR [12,14]. The urgency of tackling the mounting threat of AMR has resulted in a recent report by the Prime Minister’s Chief Science Advisor reflecting on the abovementioned objectives and assessing the progress made in each area [14].

**Figure 1:**
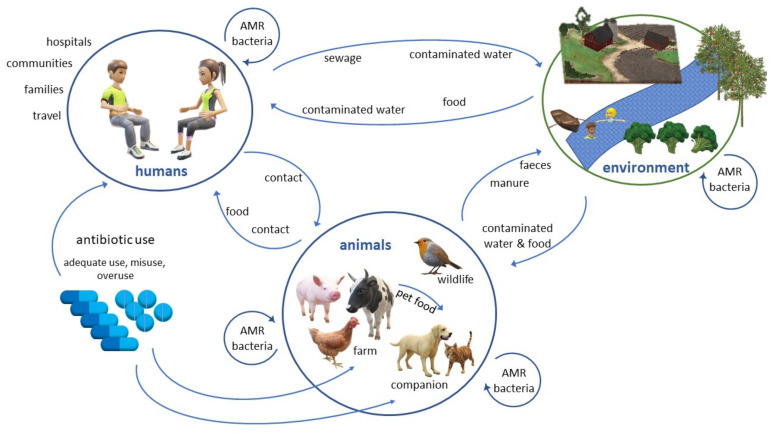
Schematic of potential transmission pathways of AMR bacteria between human, environmental and animal reservoirs.

Human, animal and environmental reservoirs contribute to the epidemiology of AMR, and transmission pathways are plentiful, both within and across the human, animal and environmental spaces (Figure 1) [15]. AMR transmission occurs via exposure to pathogenic or non-pathogenic microorganisms carrying AMR genes. These microorganisms may be either carriers (which play a role in the spread of AMR but cannot colonise the human or animal body) or vectors (able to colonise and may or may not cause disease) [16]. Pathogenic resistant bacteria may cause disease in humans or animals which can be difficult to treat. Non-pathogenic bacteria may pass on resistance genes to other bacterial species, including pathogens. Potential exposure routes to environmental AMR for humans include consumption/ingestion, inhalation or direct contact with a source [17]. Sources include environmental water (surface water used for recreation, drinking water, water used for crop irrigation, and domestic, hospital and industrial wastewater), air (may also contain dust or water droplets), contact with animals and consumption of contaminated food. Certain occupations may have an increased risk of exposure to AMR determinants, including professions working at a slaughterhouse [18], a wastewater treatment plant (WWTP) [19] or with farm animals [20]. People residing in proximity to these workplaces may also have an increased risk of exposure [19]. The most important pathway for environmental transmission of AMR is likely water contaminated with human and/or animal waste [21]. Humans use surface water as an important source for drinking water, irrigation of crops, stock water supplies and recreational activities. Livestock, pets and wild animals may also be exposed to AMR bacteria by drinking or foraging in contaminated water (Figure 1).

The global AMR crisis has led to a considerable increase in the number of studies investigating AMR in all three One Health-related areas; however, efforts have focused on human health and animal health and largely neglected environmental health [22,23,24,25,26]. Antimicrobial consumption in humans and animals and the dissemination of AMR are quite distinct to each culture and/or country [6]. NZ as a country is in a unique position, being isolated geographically but highly connected with the rest of the world by travel, trade and migration. Here, we aimed to provide a summary of the current knowledge on AMR in New Zealand, addressing all three components of the One Health triad with a particular focus on environmental AMR, and identify knowledge gaps to help develop research strategies towards mitigating AMR in the environment. The scope of this review is limited to antimicrobials—with a focus on antibacterial drugs—used as human and veterinary medicines.

## 2. Human Use of Antimicrobials in New Zealand

In NZ, human antimicrobial consumption is relatively high compared to similar countries, possibly due, in part, to antimicrobials being commonly prescribed for seasonal viral respiratory conditions in which antimicrobial treatments have no benefit [27,28]. From 2006 to 2014, the total antimicrobial consumption increased by 50%: from 17.3 defined daily doses per 1000 population per day (DID) to 25.8 DID in 2014 [28]. In 2014, the most dispensed individual antimicrobials were doxycycline (6.4 DID) and amoxicillin (6.4 DID), accounting for 49.3% of the total consumption, followed by amoxicillin/clavulanate (4.6 DID) and flucloxacillin (1.7 DID). Compared to the 29 European countries that participated in antimicrobial surveillance (European Surveillance of Antimicrobial Consumption Network; ESAC-Net) in 2013, the total antimicrobial consumption in NZ was higher than in 22 of these countries. However, a recent article highlighted that community prescription of antimicrobials in NZ has been dropping each year since 2015, with an average annual reduction of 4.6% [29]. This downward trend in community prescription of antimicrobials may reflect efforts to reduce antimicrobial use [29]. Between 2015 and 2018, the largest annual reductions were recorded for amoxicillin/clavulanate (−9.4%), fluoroquinolones (−7.7%) and macrolides (−6.7%); there was also a reduction in the use of amoxicillin (−3.6%) and tetracyclines (−3.1%), while the use of flucloxacillin was essentially unchanged (−0.4%) [29]. Even with these reductions, the total NZ community dispensing rate in 2018 (22.5 DID) was still higher compared with countries such as the Netherlands (10.05 DID), Denmark (13.98 DID) and the UK (18.2 DID) [29]. In addition, prescription of topical antimicrobials for nonindicated skin conditions may contribute to resistance development [30]. Between 1993 and 2012, dispensing of the topical antimicrobial fusidic acid increased from approximately 0.2 to 4 community dispensations per 1000 populationper month while dispensing of mupirocin decreased from about 3 to 2 community dispensations per 1000 population per month. A clear seasonal pattern was observed with dispensing rates, highest in summer and autumn [31]. A recent NZ study compared antimicrobial dispensing before and after public health interventions were introduced in 2020 to reduce the spread of COVID-19 [32]. Duffy et al. [32] found antimicrobial dispensing reduced by 36% during COVID alert levels 3 and 4 (lockdown). These reductions were mainly seen for antimicrobials used to treat respiratory and urinary tract infections—but hospital admissions due to these infections did not increase. The authors suggest that “countries with high rates of antimicrobial use could significantly reduce their use without an increase in morbidity” [32]. It should be noted, however, that while changes in service delivery and barriers to accessing primary care would have contributed to reduced antimicrobial usage during lockdowns, it is likely that there was also a decrease in the number of infections due to reduced social interactions [33].

Whilst there appears to be an effort to reduce human antimicrobial use in NZ over the past 5–10 years, there is still a need for further reduction. The recent research by Duffy et al. [32] suggests that a reduction of antimicrobial use is unlikely to result in adverse health outcomes but will rather improve health outcomes in the long term by reducing AMR.

## 3. Veterinary Use of Antimicrobials in New Zealand

NZ is one of the countries with the lowest use of antimicrobials to treat animals in the Organisation for Economic Co-operation and Development (OECD) [34,35]. In 2012, NZ ranked third lowest with 9.4 mg of the active ingredient per kg of biomass in food-producing animals after Norway (3.8 mg) and Iceland (5.9 mg) [35]. Unlike many other countries where the use and misuse of therapeutic and subtherapeutic doses of antimicrobials in agriculture is common practice [36], in NZ the use of antimicrobials as growth promotors is not permitted, and prophylactic use is only permitted with a prescription by a veterinarian. It has been shown that prophylactic antimicrobial use in animals results in heightened selective pressure and a subsequent increase in antimicrobial-resistant bacteria (ARB) [37,38,39,40]. Another reason for low antimicrobial use in NZ is that animal husbandry systems for the main food-producing animals, such as sheep and beef cattle, are relatively low in intensity and the use data are averaged across all sectors. 

However, some sectors use intensive farming systems or are moving towards intensification, which may be contributing to an increase in antimicrobial use. Between 2004 and 2015, the total sales of antimicrobials for agricultural use in NZ increased by about 2.5% per year, and by another 3% from 2016 to 2017 [35,41]. The pig, poultry and dairy cattle industries are the biggest users of antimicrobials in NZ agriculture [41,42]. In these more intensive farming systems, animals live in much closer proximity, resulting in higher rates of disease and a greater need for antimicrobials. More recently, however, there has been a decline in total sales of antimicrobials for animal use, falling 4% in 2018 and a further 10.8% in 2019 due to the effort to reduce antimicrobial use across the veterinary and production animal sectors [43]. 

While sales data can be used to estimate antimicrobial use, they may be misleading as farmers can receive bulk prescriptions, and some veterinary antimicrobials are not species-specific. In 2015, the NZ Veterinary Association (NZVA) launched the profession’s aspirational goal that “by 2030 NZ Inc. will not need antimicrobials for the maintenance of animal health and wellness” [44]. The NZVA is the first veterinary organisation in the world to make this goal explicit [44,45]. This includes, for example, prescribing dry cow therapy (treatment of cows at the end of lactation) only for cows with existing infections and not as a preventive practice [46,47,48]. About 85% of antimicrobial usage in cattle in NZ is due to mastitis management [49,50,51]. Recent data show a steady decline in whole-herd antimicrobial treatment towards targeted treatment [47]. In addition to aspirational goals, regulatory controls limiting the prescription of antimicrobials by veterinarians and ongoing investment by the Government and industry in initiatives to limit AMR are required. NZ sales data on antimicrobials used as agricultural compounds have been collected by the NZ Ministry for Primary Industries (MPI) since 2004. In 2019, the total sales decreased by 10.8% (active ingredient by weight) compared to the previous year. Antimicrobials belonging to the classes polypeptides (bacitracin), penicillins and clavulanic acid, macrolides and tetracyclines were the most commonly used in veterinary medicine. 

Antimicrobial use for companion and nonproduction animals was at 3.0% of the total NZ antimicrobial sales in 2019 [41]. Antimicrobial classes sold for use in companion animals were mainly penicillins and clavulanic acid, first- and second-generation cephalosporins and amphenicols. A 2012 study reported on 393 veterinarians’ antimicrobial prescriptions for 1799 bacterial infections in companion animals: mainly amoxicillin/clavulanic acid (48%), cephalexin (31%) and fluoroquinolones (11%) were prescribed [52]. Horticultural use of antimicrobials in NZ accounted for about 2.3% of the total antimicrobial sales in 2019, with two aminoglycoside-based products registered. The use of antimicrobials in aquaculture is common practice in many countries such as Vietnam, China and Bangladesh, but in NZ, no antimicrobials are currently registered for use in aquaculture [53,54,55].

In contrast to the human consumption of antimicrobials, veterinary use has been low, and concerted efforts have been made to control the use of antimicrobials in NZ compared to other OECD countries. It will be important to maintain this controlled approach in agriculture and aquaculture in the future.

## 4. Antimicrobial Residues in the Environment

Antimicrobials are used in community and hospital settings, veterinary clinics and on farms and for agriculture purposes, and thus these compounds are continuously released into the environment. Pathways for antimicrobial residues into the environment include discharges from WWTPs, including hospital sewage, antimicrobial manufacturing plants and agricultural wastes such as manure.

Antimicrobials are only partly metabolised by humans and animals, and thus a certain proportion is excreted as the active parent chemical in faeces and urine, contaminating wastewater and manure [56]. Some antimicrobial metabolites may be bioactive, or they may be transformed back into the parent compound or another bioactive substance [57,58]. Consequently, WWTPs are considered to be among the main “hot spots” of potential evolution and spread of AMR into the environment via different disposal routes for effluents and solids [59]. Removal rates for antimicrobials at WWTPs were found to range from zero to almost 100% and depend on the chemical characteristics of the compound and the operating conditions of the treatment system [59,60]. In NZ, no information on removal rates of antimicrobials (or AMR bacteria or genes) has been published yet. Due to the huge impact of site-specific conditions on removal rates and the high variability of treatment systems within NZ, it is not possible to extrapolate results from comparable countries.

Antimicrobials may be discharged in the environment via medicine manufacturing sites. In NZ, there are five medicine manufacturing sites that are licensed for production of antimicrobials and preparations of antimicrobials [61]. If and how much these production facilities contribute to concentrations of antimicrobials in the environment is currently unclear. Internationally, discharges from the pharmaceutical industry have been found to release higher antimicrobial concentrations into the local environment than other pollution sources [62,63,64]. Pollution from antimicrobial manufacturing sites has been mostly reported for Asia, with limited information available for Europe and other countries. Generally, due to much higher local concentrations in manufacturing effluents, the risks differ from those posed by municipal discharges [62]. Numerous effects on the biota have been reported, including resistance development and taxonomy changes in bacteria [65,66,67], immobility of water fleas [68], changed gene expression in fish [69] and stunted growth and changed behaviour in frogs and fish [62,70].

Antimicrobials in the natural environment are of concern as they not only deteriorate environmental or water quality, but also impact the natural communities present. Their presence and persistence can affect all trophic levels, from soil microbes to plants, and thus food production. Even low, subinhibitory concentrations of antimicrobials have been shown to affect several physiological reactions in microorganisms: changes in transcription levels [71]; initiation of conjugation [72]; and changes in the soil microbial community structure [73]. The persistence of antimicrobials in soil is highly variable, ranging from a few days to months [74,75]. In addition to soil texture, low temperatures and low light exposure play key roles in the fate of antimicrobials in soil environments [76,77].

There are concerns that antimicrobials in the environment exert selection pressure and add to the evolution and dissemination of AMR [78,79]. However, Karkman et al. suggest that the presence of both resistance genes and antimicrobials in wastewater and wastewater-polluted environments relates to faecal pollution levels and not necessarily to the selection pressure occurring in these environments [80]. Selective concentrations are thought to be well below minimal inhibitory concentrations (MICs), which are those completely inhibiting bacterial growth [81,82,83]. Predicted no-effect concentrations (PNECs) could be used as a measure to determine which concentrations of antimicrobials present a risk and which concentrations are unlikely to induce resistance evolution and may be regarded as “safe” [84]. It has been proposed that PNECs for 111 common antimicrobials range between 8 ng/L and 64 μg/L [84]. Murray, et al. [85] recently developed a framework for environmental risk assessments of antimicrobials with the aim to ensure discharges are safe—both regarding potentially contributing to resistance development and other environmental impacts.

There are few published studies investigating the presence and concentration of antimicrobials in NZ environmental or wastewater samples. To the best of our knowledge, only one preliminary study investigating pharmaceuticals, including ten antimicrobials, in the NZ environment (marine sediments at 13 locations around Auckland) has been conducted [86]. Four antimicrobials were above the limit of quantification: clarithromycin (range, 0.82–2.98 ngg^−1^), roxithromycin (range, 0.48–3.73 ngg^−1^), sulfamethazine (0.44 ngg^−1^, one site) and trimethoprim (range, 0.07–0.88 ngg^−1^). According to Bengtsson-Palme and Larsson [84], the measured concentrations are well below the PNEC. Studies are underway to close the knowledge gaps around key antimicrobials in NZ raw sewage and their fate throughout WWTPs [87].

To understand the long-term impacts on the environment, especially those related to effluent and wastewater disposal, more data are required. Consideration needs to be taken of both the immediate and cumulative effects across all trophic levels and the potential impacts on human health through the food chain and other transmission pathways. 

## 5. Antimicrobial Resistance in Humans and the Clinical Environment

Resistance to many common antimicrobials is endemic in NZ, in both community and healthcare settings [88]. While NZ is isolated geographically, it is highly connected with the rest of the world by travel and migration leading to the import of resistant pathogens from other countries. Pathogens with resistance to antimicrobial classes such as penicillins, third-generation cephalosporins and fluoroquinolones are often found in NZ hospitals and, with increasing frequency, in the community [88,89,90,91]. Surveillance is one of the key components to fight emergence and spread of AMR to identify priority areas for intervention and monitor their impact, to inform policy makers and to develop suitable guidelines [28]. In NZ, public health surveillance for antimicrobial resistance is conducted by the Institute of Environmental Science and Research Ltd. using EUCAST interpretation standards, if available. Reports are available online: https://surv.esr.cri.nz/antimicrobial/antimicrobial_resistance.php (accessed on 20 April 2022). Antibiograms from most NZ diagnostic laboratories, who almost all use EUCAST interpretation standards, are available online: https://www.nzmn.org.nz/antibiograms/ (accessed on 20 April 2022).

There are several key groups of AMR bacteria of significance to human health in NZ. The increased appearance of Enterobacterales (recent taxonomic changes have narrowed the definition of the family Enterobacteriaceae; some genera previously included in the family Enterobacteriaceae (e.g., *Hafnia, Morganella*, *Proteus*, *Providencia*, *Serratia* and *Yersinia*) are now included in other families in the order Enterobacterales; we, therefore, now use the order name Enterobacterales to cover the genera previously included in the family Enterobacteriaceae) with production of carbapenemases conferring resistance to carbapenems (e.g., meropenem, imipenem) or production of extended-spectrum beta-lactamases (ESBLs) leading to resistance against third-generation cephalosporins (e.g., ceftazidime) is concerning both in NZ and worldwide [92,93]. Both carbapenemase-producing and ESBL-producing Enterobacterales are included in the World Health Organisation’s (WHO) priority pathogens list with priority 1 “Critical” [94]. 

Carbapenemase-producing organisms isolated from humans are continually monitored in NZ with hospital and community laboratories requested to refer all isolates to the national Antibiotic Reference Laboratory at ESR for confirmation and further characterisation. The majority of reported infections with carbapenemase-producing Enterobacterales (CPE) in NZ are associated with a history of international travel. However, there is increasing transmission reported within NZ and CPE in patients with no overseas travel history. In 2019, travel history was reported for 87 of the 104 CPE patients, with 79% reporting overseas travel as the likely place of infection. In 2020, travel history was recorded for 66 of the 80 CPE patients, with 80% reporting overseas travel as the likely place of infection. Over the last 10 years, the number of patients diagnosed with CPE has been increasing steadily (with the exception of 2020, likely due to travel restrictions due to COVID-19) [95], with types mostly belonging to New Delhi metallo-β-lactamases (NDM) and OXA-48-like carbapenemases. Sometimes more than one class of carbapenemase is identified in individual CPE isolates. Reports on the confirmed CPE isolates since the first isolate was identified in 2009 are available online (https://surv.esr.cri.nz/antimicrobial/AccqEnterobacteriaceae.php/ (accessed on 20 April 2022)).

Numbers of ESBL-producing isolates from clinical infections have reached a level where continuous surveillance is no longer undertaken at the national level. Since surveillance of ESBL-producing isolates started in NZ in 1996, the encountered types of ESBLs have shifted from TEM- or SHV-type ESBLs to CTX-M-type ESBLs in the 2000s and 2010s [1,2]. This endemic occurrence of ESBL-producing Enterobacterales complicates treatment of infections, particularly community-acquired urinary tract infections. Reports on the confirmed ESBL isolates since 1996 are available online (https://surv.esr.cri.nz/antimicrobial/esbl.php/ (accessed on 20 April 2022)).

Carbapenem-resistant *Pseudomonas aeruginosa* and *Acinetobacter baumannii* are also included in the WHO’s priority pathogens list with priority 1 “Critical” [94]. In NZ, hospital and community laboratories are requested to refer all possible carbapenemase-producing *P. aeruginosa* and, since late 2021, all carbapenem-resistant *A. baumannii* to ESR for confirmation and further characterisation. Carbapenemase-producing *P. aeruginosa* were first found in NZ in 2009. The numbers of carbapenemase-producing *P. aeruginosa* remain much lower than the numbers of CPE, with under 40 isolates obtained between 2009 and 2020 inclusive (unpublished data). In contrast to other countries, the threat of a carbapenem-resistant *P. aeruginosa* health care-associated infection is currently very low in NZ [95,96]. 

Methicillin-resistant *Staphylococcus aureus* (MRSA) is included in the WHO’s priority pathogens list with priority 2 “High” [94]. The national surveillance of MRSA in NZ has changed over the last two decades. Initially, all MRSA were referred to ESR for characterisation. Between 2000 and 2015, annual surveys provided information on the epidemiology of MRSA in NZ, which included all MRSA isolates (clinical isolates and isolates for screening purposes) in a one-month period each year. Due to increasing numbers of MRSA isolates thereafter, biannual surveys were implemented, involving only isolates from clinical specimens [97]. In 2017, the vast majority of MRSA in NZ were acquired in the community (89%), similar to *S. aureus* infections in NZ [97]. The rate of MRSA infections has remained relatively stable between 2014 and 2017, with 18.7 and 19.9 patients with MRSA per 100,000 population, respectively. No national survey has been published since 2017. Increasing dispensing of the topical antimicrobial fusidic acid between 1993 and 2012 was concurrent with increasing fusidic acid resistance in *S. aureus* isolates (from 17% in 1999 to 29% in 2013) [31]. Similarly, a dramatic increase in fusidic acid resistance among MRSA (from 7.8% in 2003 to 37.4% in 2012) supports the hypothesis that high usage of fusidic acid in the NZ community drove fusidic acid resistance in *S. aureus* [31,98].

Vancomycin-resistant enterococci (VRE)—primarily *Enterococcus faecium* and *E. faecalis*—are associated with serious multidrug-resistant infections, and *E. faecium* is also included in the WHO’s priority pathogens list with priority 2 “High” [94]. In NZ, all hospital and community laboratories are requested to refer VRE isolates to ESR for confirmation and further characterisation. Since 2011, the number of patients with VRE has ranged between 25 (2011) and 133 (2014) [99]. The most common genotypes causing vancomycin resistance are *vanA* and *vanB* [100]. In NZ, the prevalence of *vanA E. faecium* has been increasing since 2015, which is concerning because *vanA* VRE are resistant to both vancomycin and teicoplanin while *vanB* VRE are generally susceptible to teicoplanin. While the primary transmission of VRE is thought to occur in hospitals [101]—through person-to-person contact or contaminated fomites—potential zoonotic transmission has been suggested [102]. 

Careful consideration needs to be taken when prescribing antimicrobials as there is strong evidence that overprescribing and reliance on one antimicrobial drives resistance over time. Antimicrobial resistance in the clinical setting is well-monitored in NZ; however, there are still knowledge gaps regarding the prevalence of resistance in the community as well as transmission routes, particularly environmental and zoonotic transmission pathways.

## 6. Antimicrobial Resistance in Animals

The available data suggest that prevalence of AMR in animals in NZ is relatively low [12]. Studies in NZ have measured the prevalence of AMR among *Campylobacter*, *E. coli*, *Enterococcus*, *Salmonella, Staphylococcus*, *Streptococcus* and coagulase-negative staphylococci (CNS) isolates in food-producing and farm animals (including calves, cattle, pigs, poultry and horses) and in milk, as well as among *E. coli*, other Enterobacterales, *Staphylococcus* spp. and CNS in companion animals (Table 1) [90,103,104,105,106,107,108,109,110,111,112,113,114,115,116]. Interpretation standards used in the individual studies are included in Table 1.

### 6.1. AMR in Livestock

A 2009/2010 NZ survey of AMR bacteria present in food and food animals [103] reported that about 40% of *E. coli* isolates obtained from young calves (carcass swabs) were resistant to streptomycin, sulfamethoxazole or tetracycline. None of the *E. coli* isolates was resistant to the tested third-generation cephalosporins (cefotaxime and ceftiofur), the fluoroquinolone ciprofloxacin or gentamicin and none of the isolates produced ESBLs. Some *Enterococcus faecalis* isolates were resistant to streptomycin (36%) and tetracycline (55%). Similar findings have been reported for cattle, with 53% of *E. coli* resistant to streptomycin and 47% resistant to tetracycline [115]. Likewise, 76% and 32% of *Enterococcus* spp. were resistant to streptomycin and tetracycline, respectively. Rushton-Green et al. [117] investigated vancomycin-resistant enterococci isolated from humans and poultry between 1998 and 2009. *E. faecium* lineages did not show a correlation between human and animal isolates, whereas one *E. faecalis* lineage (ST108) was highly prevalent in both human and animal isolates for several years after avoparcin use was discontinued and is indicated to have persisted and resurfaced again as late as in 2017 [117]. 

A recent review on AMR bacteria in dairy cows concluded that there is no evidence that the use of antimicrobials in NZ has resulted in the emergence of multidrug-resistant pathogens [118]. However, when considering international data, the authors saw a potential for increasing AMR in NZ dairy cows due to the use of antimicrobials, especially of third- and fourth-generation cephalosporins. A review [119] of ESBL-producing Enterobacterales in dairy farm environments discussed the role of dairy farming in the prevalence and spread of AMR from the NZ perspective. To date, cross-sectional surveys of NZ dairy farms have found a low prevalence of ESBL-producing Enterobacterales, with only one farm identified as ESBL-positive during spring but 27% of farms being positive for AmpC hyperproducing *E. coli* [107,109]. AmpC beta-lactamases are clinically important cephalosporinases encoded on the chromosomes of some Enterobacterales and on transmissible plasmids [120]. Worldwide, ESBL-producing Enterobacterales have been detected in a wide range of food products, including cheese, raw milk, beef and poultry meat, veal calves and on carcasses [121,122,123,124,125]. This highlights the potential transmission of AMR microorganisms to humans via the food chain. 

Testing of *Campylobacter* spp. isolates from an NZ beef survey [115] revealed that all the isolates were susceptible to the seven antimicrobials tested. Another NZ study that tested the faeces of dairy cattle, beef cattle, sheep and pigs for resistant *Campylobacter* spp. found that all the isolates were susceptible to the antimicrobials tested (erythromycin, ciprofloxacin, nalidixic acid, tetracycline) with the exception of five isolates derived from pig offal which were resistant to erythromycin [126]. However, in 2014, a *C. jejuni* lineage (ST6964) was identified in NZ poultry that was resistant to both fluoroquinolones and tetracycline [110]. This lineage was also associated with human infections and highlights the interconnectedness of humans and animals [110]. 

Internationally, there is a high incidence of MRSA in livestock, particularly in pigs and poultry. Transmission events have been inferred between livestock and humans, predominantly associated with pig farms [127,128]. In NZ, livestock-associated MRSA has been found in clinical isolates from people working at pig farms or in abattoirs [97]. In NZ, MRSA in milk appears to be very rare [106,129]. However, despite the low use of antimicrobials at NZ dairy farms, there has been an increase in the incidence of mastitis-associated *Streptococcus uberis* resistant to beta-lactams [130]. McDougall et al. [111] found a low prevalence of resistance against erythromycin and tetracycline in *S. aureus*, *S. uberis* and CNS isolates from milk but a high prevalence of resistance against oxacillin in *S. aureus* isolates (35%). 

### 6.2. AMR in Companion Animals

NZ studies suggest the prevalence of resistant bacteria in companion animals is higher compared with livestock. In one study, approximately 4% and 7% of the cats and dogs, respectively, carried ESBL- or AmpC-producing *E. coli* [112], while in another study, about 17% of the cats and 33% of the dogs carried ESBL- or AmpC-producing *Enterobacteriaceae* [90]. In NZ dogs, McMeekin et al. [114] found mostly low rates of cephalothin, enrofloxacin and clindamycin resistance in *E. coli*, *Staphylococcus pseudintermedius* and CNS isolates while Nisa et al. [131] found methicillin resistance in 66 out of the 176 *Staphylococcus pseudintermedius* isolates. The proximity of companion animals to humans is both a concern and a possible explanation for a higher prevalence of AMR bacteria in pets compared to livestock.

The low usage of antimicrobials in veterinary and agricultural practice in NZ has resulted in low rates of antimicrobial resistance. The NZVA’s aspirational goal to reduce unnecessary antimicrobial use positions NZ well to keep resistance rates low in animal husbandry and enables further reductions. A potential zoonotic transmission from animals (including pets) to humans needs further investigation.

**Table 1:** Antimicrobial resistance in New Zealand farm and companion animals.

Host Species	Bacterial Species	Sample Type	Resistance Phenotype	Prevalence	Method	Year of Sampling	Reference
Poultry	*C. jejuni*	Carcass	Fluoroquinolone Tetracycline	10/72 (13.9%) 25/72 (34.7%) carcasses	Disc diffusion, CLSI	2014 and 2015, respectively	[110]
	*E. coli* *C. jejuni*	Carcass rinsates	Gentamicin Tetracycline Erythromycin	6/400 (1.5%) 18/400 (4.4%) isolates ^b^ 1/200 (0.5%) isolates ^b,d^	Disc diffusion, CLSI Disc diffusion (no standard used)	2005–2006	[104]
	*E. coli* *C. jejuni*	Carcass rinsates	Cefoxitin Tetracycline Ciprofloxacin Tetracycline	3/909 (0.3%) 109/909 (12.1%) Isolates ^a,b,c^ 8/344 (2.3%) 1/344 (0.3%) isolates ^c^	Broth microdilution plates, CLSI	2009–2010	[103]
Pigs	*E. coli*	Faeces	Gentamicin Tetracycline	2/142 (1.4%) 61/142 (43%) isolates ^b^	Disc diffusion, CLSI	March–October 2001	[116]
	*E. coli*	Carcass swabs	Cefoxitin Tetracycline	12/909 (1.3%) 440/909 (48.5%) isolates	Broth microdilution plates, CLSI	2009–2010	[103]
Dairy cattle	*E. coli*	Faeces	Putative hyperproducing AmpC	11/78 (14.1%) pooled faeces from 7/26 (26.9%) dairy farms	Disc diffusion, EUCAST	May–July 2017	[109]
	*E. coli*	Faeces	ESBLs	1/116 (0.69%) pooled faeces from 1/15 (6.7%) dairy farms	Disc diffusion, EUCAST	August 2016–May 2017	[107]
	*S. aureus*	Clinical or subclinical mastitis milk	Cefoxitin	1/50 (2%) isolates	Disc diffusion, CLSI	October 2015–January 2016	[132]
	*S. aureus*	Milk	Erythromycin Oxacillin	4/320 (1.2%) 112/320 (34.9%) isolates	Broth microdilution plates, CLSI	September 2017–January 2018	[111]
Beef	*E. coli* *S. aureus*	Clinical isolates	Tetracycline Oxacillin	14/30 (46.7%) 1/6 (16.7%) isolates ^b,d^	Disc diffusion, CLSI	2003–2016	[89]
Calves	*E. coli* *C. jejuni*	Carcass swabs	Cefoxitin Tetracycline Ciprofloxacin Tetracycline	9/909 (1%) 370/909 (40.7%) isolates ^a,b,c^ 8/344 (2.3%), 1/344 (0.3%) isolates ^c^	Broth microdilution plates, CLSI	2009–2010	[103]
Companion animals	Enterobacteriaceae	Faeces	ESBLs and/or plasmid-mediated AmpC	6/18 (33.3%) dogs 3/18 (16.7%) cats	Disc diffusion, EUCAST	September 2015–September 2017	[90]
	*E. coli*	Faeces	ESBLs and/or plasmid-mediated AmpC	25/361 (6.9%) dogs 10/225 (4.4%) cats	Disc diffusion, CLSI	June 2021–June 2013	[112]
Dogs	*E. coli*	Clinical urine samples	Cephalothin Enrofloxacin Clindamycin	91/508 (17.9%) 9/500 (1.8%) 165/500 (32.5%) isolates	Disc diffusion, CLSI	2012	[114]
Horses	*E. coli*		Ceftiofur Gentamicin Tetracycline	11/24 (45.8%) 6/26 (23.1%) 16/25 (64%) isolates ^b^	Disc diffusion, CLSI	2004–2014	[113]

^a^ No resistance to cefotaxime; ^b^ no resistance to ciprofloxacin or enrofloxacin; ^c^ no resistance to gentamicin; ^d^ no resistance to tetracycline. EUCAST, European Committee on Antimicrobial Susceptibility Testing; CLSI, Clinical Laboratory Standards Institute.

## 7. Antimicrobial Resistance in the Environment

Studies suggest the main source for environmental dissemination of antimicrobials, AMR bacteria and genes is surface water predominantly impacted by human and/or animal waste and, to a lesser extent, waste application to land (Figure 1). Whilst an increasing number of these studies investigating AMR in the environment have been published worldwide, surveys on AMR in NZ’s environment are limited. 

### 7.1. AMR at Wastewater Treatment Plants

Human sewage and effluents from WWTPs have been proposed among the main sources of environmental contamination with antimicrobial residues, ARB and AMR genes (ARG), contributing to the spread of AMR [23,59,133]. Between 40% and 90% of consumed antimicrobials are excreted and end up in sewage from households and hospital discharges [63]. As a result, antimicrobials, ARB and ARGs have been detected in wastewater samples worldwide [6,23,133,134,135,136,137], including NZ [6,87]. 

Culture-dependent and culture-independent detection methods have been used to detect ARB and ARGs conferring resistance to all classes of antimicrobials at WWTPs worldwide and in their effluents, showing that treatment efficacy is quite variable and mostly insufficient [23,137,138,139]. The prevalence of ARGs and mobile genetic elements was found to change within WWTPs from influents to effluents, with the relative abundance of most genes higher in influents [133,140]. 

Effluents from both households and hospitals contribute to the quantity and diversity of ARB and ARGs in sewage systems, but it is likely that hospital effluents only contribute a minor proportion as the total volume of residential and industrial wastewater is significantly greater [134,141,142]. Resistance genes from ARB can spread among microorganisms that are part of the resident microbial community within the WWTP and those transiting through the treatment system. Due to the high density of bacteria in wastewater systems, horizontal gene transfer among pathogenic and non-pathogenic bacteria is frequently observed [133,140,143]. Wastewater also contains antimicrobials, disinfectants, heavy metals and other organic contaminants, which can exhibit selective pressure for AMR, even in low concentrations [81,83,144]]. Mobile genetic elements frequently carry resistance genes for multiple antimicrobial compounds or resistance genes for disinfectants or metals. In these scenarios, acquired resistance to one compound may co-select for resistance against another compound [145]. 

In NZ, so far, only a few small studies have investigated the presence of ARB and ARGs in WWTP effluents or environmental waters (Table 2). Studies are underway to close knowledge gaps around antimicrobials, ARB and ARGs in raw sewage and their fate throughout the treatment system [87]. A recent international study analysed the bacterial resistome in raw human sewage from 79 sites across 60 countries, including one sample taken in Dunedin, NZ [6]. The one NZ sample was among the ones with the lowest AMR gene abundances, similar to Australia. The highest AMR gene levels were detected in African and South American countries [6]. Internationally, a wide range of published literature is available, and knowledge about AMR in wastewater has increased tremendously in the past few years. However, how applicable the findings are to NZ is unknown. Antimicrobial consumption patterns are quite distinct to each culture/country. In addition, sewage treatment systems across NZ vary considerably, ranging from very basic to modern state-of-the-art facilities. Treatment processes include trickling filters, aerated lagoons, ponds, wetlands, recirculating filters and activated sludge. Out of an estimated 323 publicly owned WWTPs in NZ (in 2021), the majority are pond-based (64%, but they only serve around 17% of the total serviced population), while the more modern plants (built in the past 20 years) primarily use activated sludge processes (18%, serve 74% of the total serviced population) [146,147]. 

Whichever treatment is used, the process is not targeted at emerging compounds (pharmaceuticals, personal care products), bacteria or their genes but aims to remove organic components, nutrients (P, N) and suspended solids. Differences in treatment plant design and operation influence the fate of ARB and ARGs in wastewater; conventional activated sludge treatment combined with advanced treatment methods—such as UV or ozonation—show improved removal of ARB and ARGs compared to activated sludge alone [137,140,148]. A recent review by Pai et al. [149] estimates the removal of ARGs to be 0.1–0.6 log units with primary treatment, 1–2 log units with secondary treatment and 0–6 log units with advanced treatment methods. Whilst tertiary treatment likely offers the highest chance of AMR removal, efficacy is variable and dependent both on the treatment choice and operational management to optimise removal efficiencies. Tertiary treatment processes include UV, ozone treatment or membrane filtration to specifically remove microorganisms. A Canadian study found that even a tertiary-level WWTP meeting all regulatory target values contributed to increased downstream concentrations of ARGs [136].

In NZ, after treatment, wastewater is discharged either to waterways (rivers or the ocean) or to land [147]. A survey undertaken in 2016 found that of the council-operated WWTPs across NZ, 57% discharged to waterways, with the remaining plants discharging to land [150]. On the population basis, this equated to 11% of the national wastewater flow discharged to land. A more recent report states that of the 318 active WWTPs across NZ, 44% discharge to rivers, 20%—to the ocean, 33%—to the land [147]. By volume, most of the treated wastewater (74%) is discharged into estuaries or the ocean. This can be attributed to the country’s largest cities being located on the coast. During heavy rainfall, stormwater may enter wastewater systems, and when these get overloaded, the mixture of untreated sewage and rainwater may be discharged through sewage overflows into streams and rivers to prevent backing up onto properties. 

In addition to municipal wastewater systems, there are about 270,000 domestic on-site wastewater management systems (OWMSs) in NZ, serving approximately 20% of the population. OWMSs discharge wastewater into the land through disposal fields, potentially transporting antimicrobials, ARB and ARGs into the environment (including waterways, neighbouring properties or roadside stormwater manholes). AMR determinants may also settle out into the OWMS sludge, which is pumped out intermittently and taken to WWTPs. The potential risk to OWMSs from AMR is twofold. The first is from antimicrobials themselves which can enter the septic tank system unchanged or in the form of active metabolites and may harm the beneficial bacteria present within the system. The second risk is co-selection for AMR due to the ongoing presence of antimicrobials leading to ARB discharge into the environment.

OWMSs mirror conventional wastewater treatment systems in removing organic compounds, nutrients (P, N) and suspended solids; however, their removal capacity for AMR is limited. There is also potentially a higher risk from septic tank systems as more concentrated inputs could occur from households using prescribed antimicrobials. Within OWMSs and the surrounding disposal fields, the function of anaerobic bacteria can be reduced, leading to increased sludge accumulation in the OWMSs and higher biological oxygen demand in the environment. The aerobic bacterial population pre-treatment and the soil environment can also be negatively impacted. With OWMSs, there is an additional risk to groundwater, which in rural communities is often used for drinking, in many cases with little to no pre-treatment. This poses a risk to human health, and the use of antimicrobials could increase the risk by reducing the efficacy of OWMSs. 

Wastewater contributes to the transmission of AMR by serving as a major environmental reservoir for AMR and by providing an ideal environment for AMR microorganisms and genes [151]. High levels of AMR bacteria and genes were detected in untreated sewage, influent and effluent samples from WWTPs and hospitals, in industrial (including pharmaceutical treatment plants) and agricultural wastewater. Consequently, increased levels of AMR bacteria and genes were detected downstream of discharges, rivers and even tap water. How long resistant bacteria and genes persist in these environments is not well-known, but given the constant discharge of very large volumes of wastewater, ongoing replenishment can be expected. Studies have demonstrated that clinically relevant bacterial species such as *Salmonella* spp. and enterotoxigenic *E. coli* can persist in environmental water for long times [152,153]. Due to the abovementioned pathways, including treated sewage discharges and untreated sewage stormwater overflows, it is likely that faecal bacteria, including ARB, ARGs and antimicrobials are released into the aquatic environment in NZ—similar to many other countries—and may present a public health risk. 

### 7.2. AMR after Waste Application to Land

Application of human and/or animal wastes to land—solids, manure, greywater or wastewater—is common practice worldwide. In NZ, application of animal wastes is widely accepted and practiced while application of treated human waste is often met with concerns. Treated wastewater in NZ is mostly discharged to waterways, but 33% is applied to land, with land disposal systems varying widely [147]. When wastewater is applied to land, it mostly occurs via high-rate methods such as constructed wetland and soakage trenches or to grazed pastures (not dairy). The remaining wastewater is applied to trees and cut-and-carry pasture harvesting systems. Depending on the level of treatment prior to discharge, there is a variable risk of pathogen transport, including ARB, to land. Biosolids produced by treatment processes are mainly disposed of via quarry rehabilitation or landfills [154]. A smaller proportion is applied to land, in forestry or serves as landfill cover. Depending on the source and pre-treatment of waste products, they can contribute pathogens, ARGs and various other contaminants to the environment.

#### 7.2.1. Animal Waste to Land

Antimicrobial use in farm environments and consequently the emergence and distribution of ARB and ARGs in farm wastes is of concern for both human and animal health. Worldwide, pathogens of human concern resistant to antimicrobials have been detected in animal manure, dairy farm environments and food products [155,156,157,158]. After administration of antimicrobials, animals excrete substantial amounts in urine and faeces (between 17% and 75% for livestock species), either unchanged or as active metabolite [159]. Application of livestock manure for fertilisation, run-off from pastures or dairy shed effluent may lead to contamination of surface water with pathogens, antimicrobials, ARB and ARGs.

Antimicrobials or their residues can persist in the soil environment and potentially accumulate in the environment by adsorption to soil particles [73,160] and thus may drive the development of AMR [78]. In the soil environment, microbial communities play a key role in AMR transmission, as do environmental factors [74,75,76,77,161]. One such factor is the soil itself [161]: in addition to the type of ARGs, the soil texture plays a key role in the persistence of ARGs. The persistence in receiving waters is also linked to the soil texture present, with ARGs more persistent in water adjacent to sandy soils compared to clay soils. A potential method of reducing the input and thus the impact of antimicrobials (or their residues), ARB and ARGs on land has been suggested where manure is treated first (e.g., composting). There have been mixed results with regard to the efficacy of composting. Some researchers have found a significant reduction in antimicrobial and ARG concentrations in composted manure [162,163,164,165,166], whereas others have found a limited efficacy or even an increase in some ARGs [163,166]. Composting conditions need to be optimised carefully (e.g., pH, temperature, maturation duration) for optimal degradation. 

There is a lack of research into the extent of antimicrobials (or their residues), ARB and ARGs entering the NZ environment from animal wastes. A report compiled by the NZ Ministries of Health and Primary Industries pointed to a lack of consistent surveillance and research on the risk to the environment from animals [12].

#### 7.2.2. Human Waste to Land

Conventional activated sludge processing at WWTPs is inefficient at removing antimicrobials which end up in sludges and biosolids that may be applied to land. Some antimicrobials, such as sulphonamides, fluoroquinolones, erythromycin and tetracycline are preferentially removed into the sludge, either by physical adsorption or enhanced adsorption through addition of flocculants [167,168,169,170]. The fate of ARB and ARGs through wastewater systems, including sludge, has been extensively studied, with the focus often on a small number of resistance genes or specific bacterial species [137,148]. Results are usually very dependent on the system studied and cannot easily be extrapolated to other WWTP and AMR determinants. Conditions at WWTPs seem to favour development and spread of AMR [143]; while there is a big shift in the microbiome throughout treatment stages [3], a reduction in some AMR determinants occurs while others have been found to become enriched [143]. This shift and preferential removal in different fractions should be considered when assessing the risk associated with the application of solids or water to land or discharge into waterways.

As mentioned in the previous section, there is a paucity of knowledge on the risk of OWMS wastewater application to land from antimicrobials or ARGs. This is also the case for biosolids application to land.

#### 7.2.3. Greywater

With the growing pressure on water supplies globally, there has been an increase in reclamation of water, including in waste streams. The use of greywater (domestic wastewater excluding wastewater from toilets) for irrigation of crops, golf courses and landscapes is becoming more common, but there is increasing concern that this could provide another AMR pathway to land [171,172,173]. In NZ, to the best of our knowledge, there is little information available regarding greywater application with respect to AMR. Zaayman [174] investigated the risk to the health of soil bacteria from the application of greywater amended with the antimicrobial triclosan. The study investigated the risk to an NZ silty clay loam from the leachate from a greywater system applied over time. A reduction in the respiration rate of bacterial biomass was observed, but it was significant only at high concentrations of triclosan (over 5000 ppm). Other indicators of soil health were investigated, and a negative impact of triclosan at much lower concentration (195 ppm) was reported. The study concluded that although the impact of triclosan on the immediate soil environment may be low, there is a high risk of triclosan being transported throughout the soil profile and below. Once there, it may be able to persist for prolonged periods of time posing a risk to the microbial fauna present. 

### 7.3. AMR in Environmental Water

AMR bacteria are present in surface water all over the world, and wastewater significantly contributes to this. A Dutch study investigated 30 water bodies and wastewater samples from five health care institutions, seven municipal WWTPs, and one airport WWTP [175]. Multidrug-resistant and ESBL-producing *E. coli* were isolated from all water sources with concentrations of multidrug-resistant *E. coli* isolates increasing in the following order: surface water (2.2 × 10^2^ cfuL^−1^), WWTP effluents (4.0 × 10^4^ cfuL^−1^), WWTP influents (1.8 × 10^7^ cfuL^−1^) and health-care wastewater (4.1 × 10^7^ cfuL^−1^). A Canadian study [136] found that ARG concentrations decreased as proximity to human-impacted areas decreased. The same study also suggested that ARGs might be ubiquitous in watersheds even without obvious pollution sources. An NZ study by Gray et al. [176] also indicates that ARB are associated with human-impacted areas. Here, ESBL-producing *E. coli* were isolated downstream of the effluent outflow, but not downstream of the land used for dairy farming. 

In NZ, a limited number of studies have investigated AMR in surface water (summarised in Table 2). A cross-sectional study carried out in NZ’s largest city, Auckland, found ESBL-producing *E. coli* from these urban waterways were genetically similar to human clinical (derived from urinary tract infections) and dog faecal isolates [177,178]. In the Canterbury region along the Waimakariri river, Schousboe et al. recorded an increase in antimicrobial-resistant *E. coli* between 2004 and 2012 [179]. Another NZ study investigated 10 AMR genes in freshwater biofilms at six sites along a river in Otago for one year [180]. Three AMR genes were detected (using polymerase chain reaction), including *vanB*, which confers resistance to vancomycin. In a spatiotemporal follow-up study of 20 sites, three of the ten AMR genes were detected in 1.3% of the biofilm samples, with more genes detected close to intensive farming areas, suggesting a moderate correlation [181]. Fish & Game NZ commissioned a study to test water and sediments of three Canterbury rivers in May and September 2018 for *E. coli* and the presence of six different genes associated with virulence and one group of AMR genes. In the September (spring) sampling, water and sediments of two rivers were positive for the *bla*_CTX-M_ genes which confer resistance against beta-lactams, including penicillins and third-generation cephalosporins [182]. Further studies investigated AMR coliforms and *E. coli* in surface water across NZ and in mussels deployed in an urban harbour and confirmed the presence of microorganisms resistant to some of the tested clinically relevant antimicrobials, including ESBL-producing *E. coli* in the water samples [183,184,185].

Whilst studies are underway and our knowledge is improving on the fate and impact of AMR in the natural environment, there are still more data needed to provide evidence of the impact on human health.

**Table 2:** Antimicrobial resistance in the New Zealand environment.

Environment ^a^	Analytical Target	Sample Type	AMR Phenotype/AMR Abundance	Prevalence/Total Gene Abundance	Method	Year of Sampling	Comments	Reference
Human sewage and WWTP effluents	Resistome analysis	Raw municipal sewage	AMR genes with the highest relative abundance: Macrolide Beta-lactam Tetracycline Aminoglycoside	AMR gene levels in NZ sewage: approximately 530 fragments per kilobase per million fragments (FPKM)	Whole sample metagenomic shotgun sequencing	2016 One sample	The study has been ongoing with more samples included from a number of NZ cities; the results are pending	[6]
	Resistome analysis	Raw municipal sewage, effluents, oxidation pond water and sediments	AMR genes with the highest relative abundance: Macrolide Beta-lactam Tetracycline Aminoglycoside	400 different AMR genes identified across all the sample types	Whole sample metagenomic shotgun sequencing	2019	The number of resistance genes decreased throughout the treatment	[87]
Environmental water	*E. coli*	Urban waterways, dog faeces	ESBL, AmpC	*n* = 31 isolates 23% ESBL 23% AmpC	Disc diffusion, CLSI	2017/2018	*E. coli* were grown on selective agars	[177,178]
	*E. coli*	Large rural river	Streptomycin Sulphafurazole Tetracycline Trimethoprim Ampicillin Chloramphenicol ^b^ Nalidixic acid ^b^ Nitrofurantoin ^b^ Cefaclor ^b^	9/63 (2004) 16/80 (2012)	Disc diffusion, CLSI	2004 and 2012	Resistant isolates were resistant to a subset of the tested antimicrobials	[179]
	*vanA, vanB, mecA, ermA, ermB, tetA, tetB, tetK, tetM, aacA-aphD*	Rural river freshwater biofilms	*ermB, vanB* and *tetB* genes were detected	In 2% of the 147 samples, AMR genes were detected, six sites/three rocks per site	PCR	2010/2011		[180]
	*vanA, vanB, mecA, ermA, ermB, tetA, tetB, tetK, tetM, aacA-aphD*	Freshwater biofilms from four waterways	*ermB*, *tetK* and *tetM* detected	1.3% overall detection, 480 samples/20 sites/three rocks per site/eight samplings	PCR	2010/2011		[180,181]
	*E. coli*	Surface water (urban and rural streams) Mussels	ESBLs Ampicillin Chloramphenicol Ciprofloxacin	N/A	Disc diffusion, CLSI	2017	Isolation of *E. coli* on selective media containing different antimicrobials	[183,184]
	*E. coli*, virulence genes, *bla*_CTX-M_	Rural river, water and sediments	*bla*_CTX-M_	*bla*_CTX-M_ present at two sites in September (water and sediments)	PCR	May and September 2018		[182]

^a^ Studies are underway to close knowledge gaps on AMR after waste application to land and AMR in wastewater (including antimicrobials, resistance genes and ESBL/AmpC- and carbapenemase-producing Gram-negative bacteria). ^b^ 2012 only. EUCAST, European Committee on Antimicrobial Susceptibility Testing; CLSI, Clinical Laboratory Standards Institute.

## 8. Summary and Conclusions

Antimicrobial resistance and antimicrobial usage in humans are relatively well-monitored and -understood in NZ, with ready access to antimicrobial dispensing data and ongoing monitoring of the key groups of clinical AMR pathogens. Human use of antimicrobials is high in NZ, and pathogens with resistance to antimicrobials are found in hospitals and the community and are increasing. However, compared to other countries, the public health burden of AMR in NZ is fairly low [12]. Some pathogen/resistance combinations that pose huge problems elsewhere are not yet common in NZ—but could be in the future. Future challenges include increased levels of resistance in many common and serious bacterial infections, resulting in increased morbidity and mortality due to reduced treatment options. Human infection with most AMR bacteria in NZ is believed to occur mostly person to person or via contaminated fomites and—to a lesser degree—through contact with (farm) animals, though AMR at NZ farms is estimated to be low overall. Companion animals have a slightly higher carriage of AMR bacteria than farm animals, which may be a concern for human health due to the close contacts between pets and owners. The role of NZ’s natural environment in AMR transmission is unclear. There is evidence of AMR in wastewater, in surface water impacted by human and/or animal waste and in sea water and mussels. Systematic monitoring of AMR determinants in waste products of pharmaceutical companies, abattoirs, intensive farming operations, municipalities and hospitals would help to identify the main sources and pathways of AMR in NZ’s natural environment. Since a range of contaminants, including biocides and heavy metals, are known to promote AMR in the environment, it is crucial to clearly establish all drivers and pathways of AMR in the environment [144]. Increased surveillance to close the outlined knowledge gaps will help to gain a better picture around the release of antimicrobials, ARB and ARGs into the environment and the potential impact on human, animal and environmental health in New Zealand.
